# Effects of propofol on the cognition and hippocampal N-methyl-D-aspartate subunits expression in an MPTP-induced Parkinson’s disease rat model

**DOI:** 10.3389/fnbeh.2025.1607421

**Published:** 2025-06-02

**Authors:** Ping Zhu, Yongyan Zhang, Hua Xu, Yu Ren

**Affiliations:** ^1^Department of Anesthesiology, Yueyang Hospital of Integrated Traditional Chinese and Western Medicine, Shanghai University of Traditional Chinese Medicine, Shanghai, China; ^2^Department of Anesthesiology, Fudan University Shanghai Cancer Center, Shanghai, China

**Keywords:** cognition, propofol, Parkinson’s disease, NMDAR, hippocampal

## Abstract

**Introduction:**

Parkinson’s disease (PD) is associated with higher risk of cognitive impairment. Until now, little is known about the effect of anesthetics on cognitive function in PD patients. The imbalance of hippocampal N-methyl-D-aspartate (NMDA) receptors NR2A/NR2B subunit ratio is reported to be associated with memory dysfunction in PD rats. The current study investigated the effects of propofol on the cognitive function and hippocampal NR2A/NR2B ratio in a 1-methyl-4-phenyl-1,2,3,6-tetrahydropyridine (MPTP)-induced PD rat model.

**Methods:**

MPTP was stereotaxically injected into the substantia nigra pars compacta of male Wistar rats. Next day (day 2), the rats in the chronic intervention groups were injected daily with either propofol (80 mg/kg/day, i.p.) or fat emulsion for 7 days (day 2–8). The rats in the acute intervention groups received propofol or fat emulsion only on day 8. Then, all the rats underwent an open field test and an inhibitory avoidance (IA) test. At last, the rats were killed for histological analysis and hippocampal NR2A and NR2B proteins and mRNA level quantification.

**Results:**

Neither acute nor chronic treatment with propofol can significantly change the impairment of locomotor activity and dopaminergic denervation of the striatum in MPTP-lesioned rats. MPTP lesioning caused IA memory impairment, which was aggravated by chronic treatment with propofol. Furthermore, chronic treatment with propofol also aggravated the imbalance of hippocampal NR2A/NR2B ratio in MPTP-lesioned rats.

**Discussion:**

The current findings indicate that chronic propofol treatment exacerbated MPTP-induced inhibitory avoidance (IA) memory impairment and aggravated the imbalance of hippocampal NR2A/NR2B ratio in MPTP-lesioned rats. Our current data demonstrate a correlation, not direct causation, between NR2A/NR2B dysregulation and cognitive impairment. Future studies should probe whether this imbalance is a driver or consequence of synaptic dysfunction.

## Introduction

For Parkinson’s disease (PD) is the second most common neurodegenerative disease with a global prevalence of more than 6 million individuals (Tolosa et al., 2021). This number corresponds to a 2.5-fold increase in prevalence over the past generation, making Parkinson’s disease one of the leading causes of neurological disability (Dorsey et al., 2018). In addition to the defining motor symptoms of PD, multiple non-motor symptoms occur; among them, cognitive impairment is common and can potentially occur at any disease stage (Aarsland et al., 2021). Twenty to thirty percent of patients with PD suffer from symptoms of dementia, named Parkinson’s disease dementia (Aarsland et al., 2005).

Cognitive impairment in PD is associated with older age (Baiano et al., 2020). Postoperative cognitive dysfunction (POCD), especially in elderly patients, has also been a distressing problem. Its occurrence may herald an increase in morbidity and mortality (Zeng et al., 2023). Until now, there is still controversy about the potential effects of general anesthetics on POCD (Zhu et al., 2024). PD patients undergoing propofol sedation may face overlapping risks with POCD (e.g., age-related vulnerability, neurodegenerative substrate, and GABA/NMDA receptor modulation by propofol) (Guan et al., 2024). And, more and more animal studies suggest that general anesthetics may both cause and mitigate existing neuropathology (Hudson and Hemmings, 2011; Sellbrant et al., 2016). Therefore, it is necessary to know whether and how general anesthetics influence the cognitive function of patients with PD.

The mechanisms underlying cognitive impairment in PD are poorly defined. In addition to dopaminergic (DAergic) degeneration, hyperactivity of the glutamatergic system is also confirmed to play a key role in the pathophysiology of cognitive impairment in PD (Hsieh et al., 2012; Campanelli et al., 2022). Excessive synthesis and release of glutamate can overstimulate N-methyl-D-aspartate (NMDA) receptors, causing calcium overload in neurons and triggering apoptotic cell death (Reiner and Levitz, 2018). Blockade of NMDA receptors has been found to be effective in the treatment of PD (Sitzia et al., 2020). The hippocampus is a key structure for learning and memory. Both structural and functional abnormalities of the hippocampus have been observed in PD (Foo et al., 2017; Kandiah et al., 2014). Hippocampal NMDA receptors are involved in the formation of cognitive functions. Both NR2A and NR2B subunits have been found to be required for hippocampal long term potentiation (LTP) (Belloso-Iguerategui et al., 2023). And, it has been reported that the imbalance of NR2A/NR2B subunit ratio in hippocampal synaptic NMDA receptors might contributes to LTP impairment and memory dysfunction in both a neurotoxic and a genetic model of PD (Costa et al., 2012).

Propofol is widely used as an intravenous anesthetic in clinical anesthesia. It has been proved to exert neuroprotective actions by inhibiting NMDA receptor-mediated calcium increase (Grasshoff and Gillessen, 2005). Chronic treatment with propofol has also been found to improve cognitive function in both aged and Alzheimer’s disease (AD) transgenic mice (Shao et al., 2014). Whether propofol exerts similar effects on cognitive function in Parkinson’s disease remains entirely unexplored. Therefore, the primary objectives of this study were to: (1) Systematically evaluate the impact of acute versus chronic propofol administration on cognitive function in MPTP-induced PD rats. (2) Determine whether these effects correlate with alterations in hippocampal NR2A and NR2B subunit expression. (3) Elucidate potential glutamatergic mechanisms underlying propofol-associated cognitive changes in PD. This study aims to provide experimental data about the effect of propofol on the PD rats model to support further research.

## Materials and methods

### Animal

This research was approved by the Institutional Animal Care and Use Committee (Shanghai Fudan University School of Medicine, Shanghai, China). A total of 60 Wistar rats (male, 400–420 g) was housed in a temperature-controlled room (21–23°C) on a 12 h light/12 h dark cycle (lights on 6:00 am–6:00 pm). Standard laboratory chow and water were available ad libitum. Behavioral testing was performed during the light cycle between 9:00 am and 1:00 pm. Rats were given 1 week to acclimatize to the facility before the start of experiments.

### Animal grouping and drug treatment

Sixty rats were randomly divided into six groups. Three groups were used for the acute intervention experiments: (i) sham + vehicle-acute, (ii) MPTP + vehicle-acute, (iii) MPTP + propofol-acute. Another three groups were used for the chronic intervention experiments: (iv) sham + vehicle − chronic, (v) MPTP + vehicle − chronic, (vi) MPTP + propofol-chronic.

MPTP is a neurotoxin with strong lipophilicity that can enter the central nervous system through the blood-brain barrier, causing a series of reactions such as oxidative stress, inflammation, and mitochondrial apoptosis, ultimately leading to damage to dopaminergic neurons in the substantia nigra. This model is widely used in PD research (Mustapha and Mat Taib, 2021). All rats were subjected to stereotaxic surgery on day 1. The rats were anesthetized for surgery using an intraperitoneal injection of sodium pentobarbital (50 mg/kg). Either MPTP (1 μmol in 2 μL, Sigma, Shanghai, China, MPTP-lesioned group) or the same volume of saline (sham group) was bilaterally injected into the substantial nigra compact (SNc) using the following coordinates: anteroposterior, −5.0 mm from bregma; mediolateral, ±2.0 mm from midline; dorsoventral, −7.7 mm from skull surface. Rats were carefully monitored after the surgery and were handled daily for 7 days (3 min/day) before receiving behavioral training.

Both sham and MPTP-lesioned rats then received the treatments detailed below. For the acute treatment, either vehicle [fat emulsion (Sigma, Shanghai, China)] or propofol (Beijing Fresenius Kabi Pharmaceutical Co., Ltd., Beijing, China) was administered for one time (80 mg/kg; i.p.) 7 days after the surgery. For the chronic treatment, either fat emulsion or propofol was administered since the second day after the surgery for consecutive 7 days (80 mg/kg; i.p, qd). The dosing regimen of propofol was based on previous report (Zhu et al., 2015). Twenty-four hours after the end of the treatments, rats were subjected to behavioral tests.

### Behavioral tests

#### Open field test

The open field test was performed 8 days after stereotaxic surgery. Each rat was placed into the center area of the open-field (ENV-520, Med Associates Inc., Vermont), and was allowed to freely explore the field for 30 min. The total distance traveled and ambulatory counts were automatically recorded by monitor system to evaluate the locomotor activity of the rats.

#### Inhibitory avoidance test

The inhibitory avoidance (IA) test was performed the day after the open field test. A shuttle-box for rats (MED-APA-D1R, Med Associates Inc., Vermont) was used to examine inhibitory avoidance learning. The box consists of two chambers, one of which is dark, and the other one is illuminated by a bulb (28 V DC, 100 mA), with a door accessible to each other. The procedures consisted of training and retention sessions. During the training session, a rat was placed into the illuminated chamber, facing away from the door, and was allowed to freely exploring the chamber for 30 s. Thereafter, the door was automatically open, making the dark chamber accessible. Once the animal crossed into the dark chamber, the door was closed, and a single foot shock (0.8 mA for 2 s) was delivered immediately. The animal was remained in the dark chamber for 30 s after the foot shock, and then was returned to its home cage. All rats successfully entered the dark chamber within 300 s during training trials, so no rats were excluded.

The retention test was conducted 24 h after the training session. During the retention test, rats were individually put into the illuminated chamber, facing away from the door. Thirty seconds later, the door was then automatically open, and the latency to step into the dark chamber was automatically recorded. The maximal observation time was 300 s.

**Figure 1:**
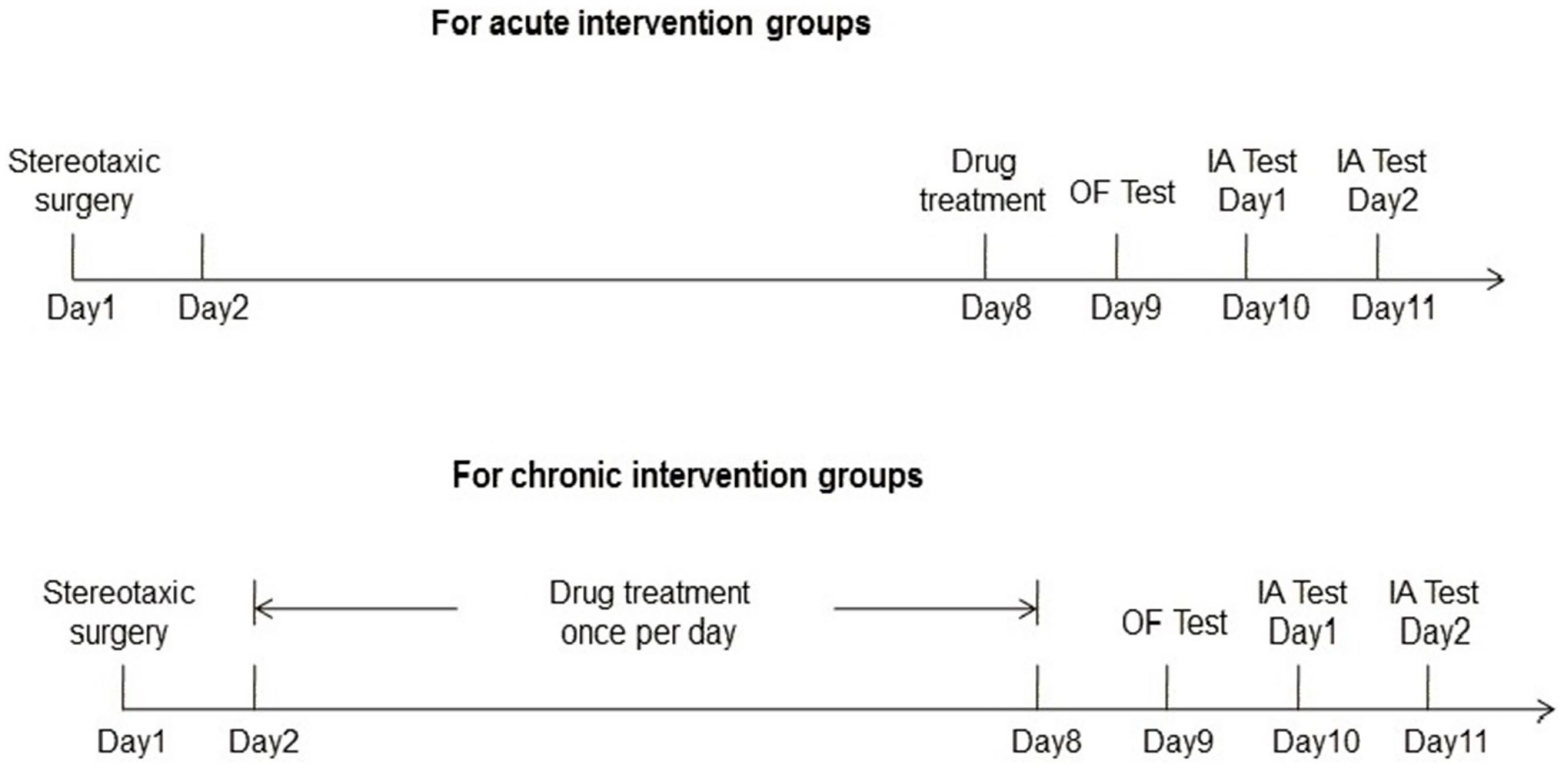
The experimental procedures of the current study.

The experimental procedures were shown in Figure 1.

### Histological characterization

After behavioral tests, half the number of rats from each group were used for this experiment. Briefly, after deeply anesthetized with sodium pentobarbital (150 mg/kg), rats were intracardially perfused with saline and then 4% paraformaldehyde (PFA) containing saturated picric acid (pH 7.4). Brains were then post-fixed with 4% PFA in 20% sucrose overnight, and paraffin embedding was processed. Coronal brain section (8 μm) was then made, based on the rat brain atlas. Sections containing area of striatum (bregma −0.20 mm) were immunostained overnight at 4°C with tyrosine hydroxylase (1:1500, AB152, Millipore). According to the work of Hsieh et al. (2012) in the striatum, the density of DAergic projections were measured by converting the TH-stained images to gray-scale. Then we measured the gray level of the area of interest and subtracted background staining measured in the non-immunoreactive corpus callosum. At last, the background-corrected optical density of the TH-reactive tissue was obtained.

### Western blot

After behavioral tests, another half of the rats from each group were used for this experiment. After deeply anesthetized with sodium pentobarbital (150 mg/kg), these rats were killed and their brains were removed. The hippocampal tissues were isolated with a spatula and immediately stored in liquid nitrogen. The expression of NR2A and NR2B in the hippocampus was examined with western blot. Briefly, 15 μg of protein lysate from each rat were separated with 10% SDS-polyacrylamide gel, and then were electronically transferred onto a PVDF membrane. Membranes were blocked in 5% BSA/TBST for 1 h at room temperature and then incubated overnight with NR2A (1:500, AB1555P, Millipore, Billerica, MA) or NR2B (1:500, AB1557P, Millipore, Billerica, MA) in 5% BSA/TBST at 4°C. After washing with TBST, the membranes were incubated with horseradish peroxidase-labeled secondary antibodies. Blotting signal was visualized with the ECL detection system (Pierce). All membranes were re-blotted to GAPDH (1:5,000, Santa Cruz Biotechnology Inc., Santa Cruz, CA), in order to normalize the loading amount. Densitometry with ImageJ analysis software was used to qualify the expression level.

### Quantitative real-time PCR

Total RNA was extracted from the rats’ hippocampal tissues with Trizol reagent (Thermo Fisher). cDNA was synthesized and quantitative real-time PCR amplification reactions were performed. Then, qRT-PCR was performed using SYBR Green Master Mix (Vazyme, China) on a Quant Studio 1 Real Time PCR system (Thermo Fisher, United States). GAPDH was used as a control. The primers used for qPCR analysis were: GAPDH forward: 5′-ACAGCAACAGGGTGGTGGAC-3′, GAPDH reverse: 5′-TTTGAGGGTGCAGCGAACTT-3′; NR2A forward: 5′-AGCCCCCTTCGTCATCGTAGA-3′; NR2A reverse: 5′-ACCCCTTGCAGCACTCTTCAC-3′; NR2B forward: 5′-TGAGACTGAGGAGCAAGAGGATGAC-3′, NR2B reverse: 5′-GCTTCTGGCACGGGACTGTATTC-3′. PCR of every sample was repeated three times, and the data were normalized to GAPDH expression and expressed as a fold change compared to the control by the 2^−ΔΔCt^ method.

### Statistical analyses

No statistical methods were used to pre-determine sample sizes, but our sample sizes are similar to those in previous reports (Zhu et al., 2023; Chen et al., 2020) and are typical of the field. Statistical analysis was performed using the SPSS 14.0 software package (SPSS, Chicago, IL), and the normality of the data were evaluated. All values were presented as the mean ± SD. Analysis of variance (one-way ANOVAs), followed by the Tukey’s *post hoc* test was used to analyze the open field test, IA test, histological, western blot and PCR. To determine whether IA test learning had occurred, paired *t*-tests were used to compare the training and retention latencies of the sham-vehicle groups. *p* < 0.05 was considered statistically significant.

## Results

### Neither acute nor chronic treatment with propofol can significantly change the impairment of locomotor activity in the MPTP-induced PD rat model

**Figure 2:**
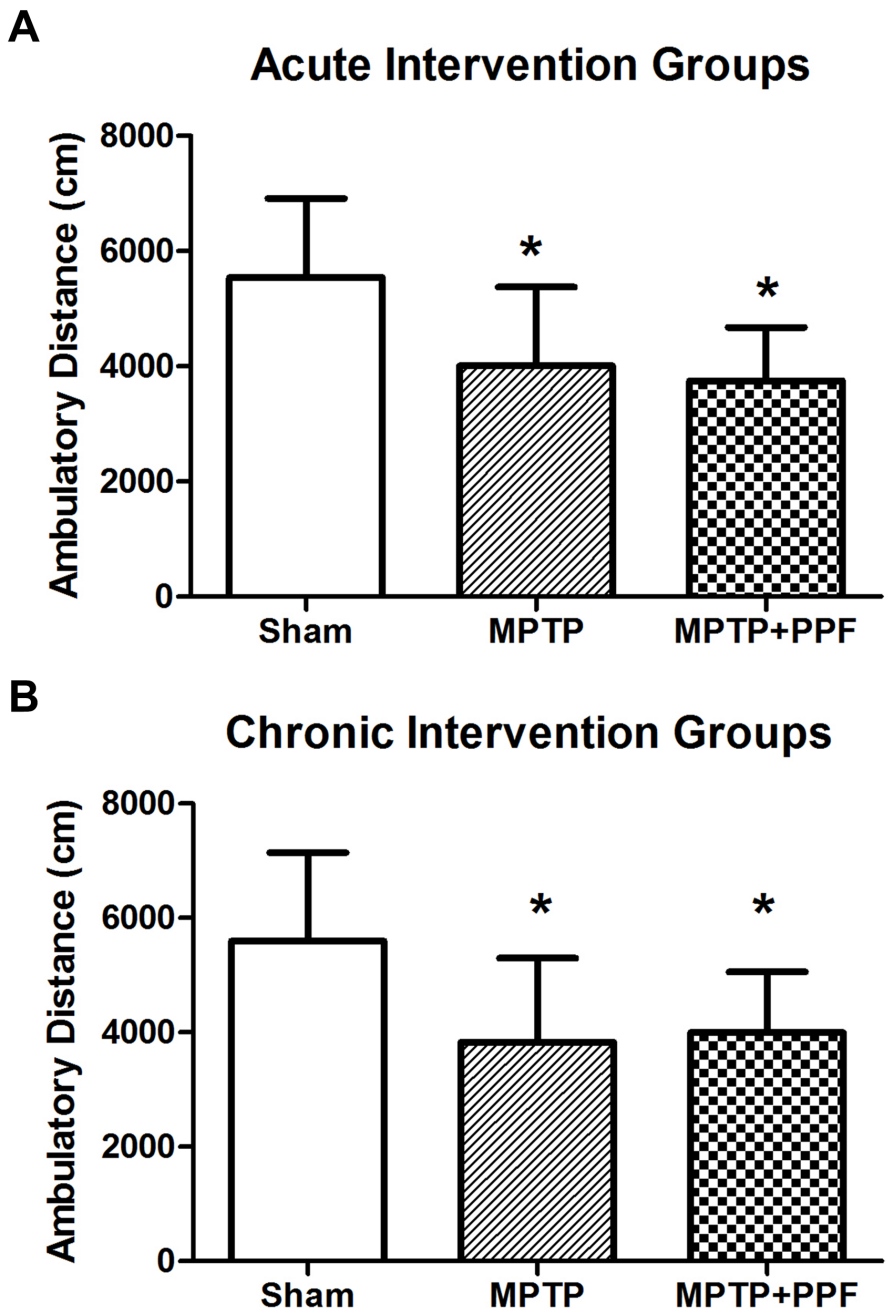
Statistical difference of the total distance traveled was compared among the acute **(A)** and chronic **(B)** intervention groups, respectively. In the open field test, total distance traveled was used to evaluate the locomotor activity. Rats-treated with MTPT showed a significantly lower activity in locomotion, compared to that in sham-lesion rats. Moreover, this deficit could not be attenuated by either the acute or chronic treatment with propofol. Data are expressed as mean ± SD, ^*^p < 0.05 compared with the control (sham) group, *n* = 10 in each group.

In the open field test, total distance traveled was used to evaluate the locomotor activity of the rats. The results of the acute and chronic intervention groups were shown in Figure 2. One-way ANOVA performed at the total distance traveled revealed a significant difference across groups (acute intervention groups: *F*_2,27_ = 12.72, *p* < 0.001; chronic intervention groups: *F*_2,27_ = 21.32, *p* < 0.001). Additional analysis with Tukey’s multiple comparisons tests (MCT) showed that both rats in the MPTP + vehicle-acute group (*n* = 10) and MPTP + propofol-acute group (*n* = 10) showed a significantly lower activity in locomotion with mean total distance traveled 4011.49 ± 1367.85 cm and 3749.06 ± 927.89 cm, respectively [*p* = 0.027, *p* = 0.009 vs. rats in the sham + vehicle-acute group (*n* = 10, 5542.47 ± 1371.06 cm), respectively]. Both rats in the MPTP + vehicle-chronic group (*n* = 10) and MPTP + propofol-chronic group (*n* = 10) showed a significantly lower activity in locomotion with mean total distance traveled 3830.44 ± 1467.42 cm and 3994.18 ± 1063.45 cm, respectively [*p* = 0.021, *p* = 0.039 vs. rats in the sham + vehicle-chronic group (*n* = 10, 5593.80 ± 1548.22 cm), respectively]. There was no difference in the locomotor activity deficit between the MPTP + vehicle groups and the MPTP + propofol groups in both acute (*p* = 0.884) and chronic (*p* = 0.962) propofol treatments.

### Chronic treatment with propofol significantly worsens the impairment of IA memory in an MPTP-induced PD rat model

**Figure 3:**
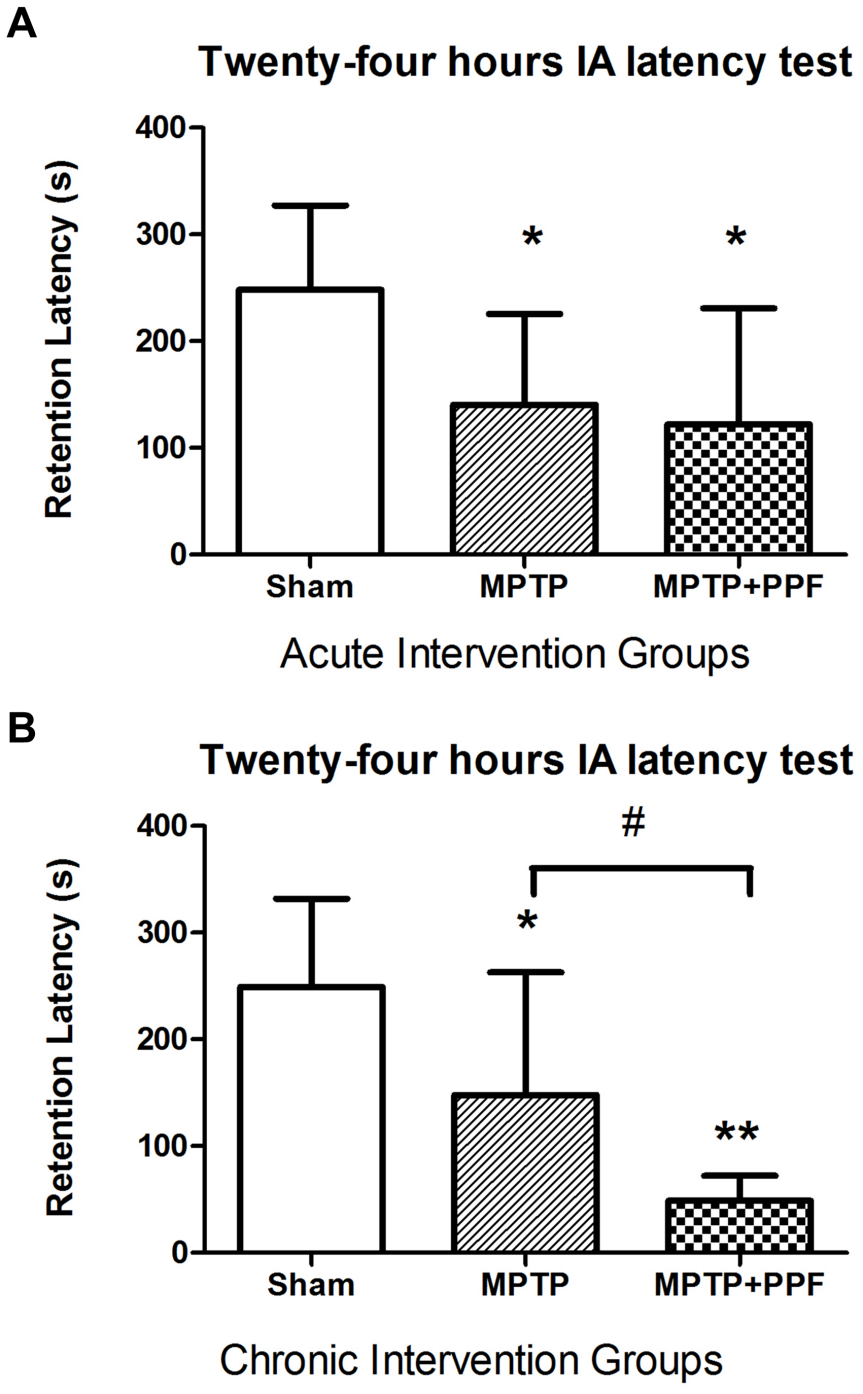
Statistical difference of the 24-h inhibitory avoidance memory retention performance compared among the acute **(A)** and chronic **(B)** intervention groups, respectively. The finding implicated that rats-treated with MTPT showed a significantly shorter step-through latency, compared to that in sham-lesion rats in both the acute and chronic intervention experiments. This deficit could not be modulated by the acute treatment with propofol. However, the chronic treatment with propofol (80 mg/kg/day for 7 days) significantly enhanced the MTPT effect on impairing the memory performance. Data are expressed as mean ± SD. ^*^p < 0.05 and ^**^p < 0.001 compared with the control (sham) group. ^#^*p* < 0.05 compared with MPTP group, *n* = 10 in each group.

The results of acute and chronic intervention groups were shown in Figure 3. A significant change in step-through latency was not found between any two groups during the training session (data not shown). Twenty-four-hours retention latencies of rats in the sham + vehicle-acute group (*n* = 10) was significantly longer than their entrance latencies during the training trial (248.11 ± 79.13 s vs. 20.81 ± 16.44 s, *p* < 0.001), indicating that the rats retained memory of the shock experience.

One-way ANOVA performed at the retention latencies revealed a significant difference across groups (acute intervention groups: *F*_2,27_ = 18.56.72, *p* < 0.001; chronic intervention groups: *F*_2,27_ = 34.75, *p* < 0.001). Additional analysis with Tukey’s MCT that both rats in the MPTP + vehicle-acute group (*n* = 10) and MPTP + propofol-acute group (*n* = 10) had obvious amnesia, with retention latencies of 140.58 ± 85.03 s and 122.02 ± 108.74 s, respectively [*p* = 0.037, *p* = 0.013 vs. rats in the sham + vehicle-acute group (*n* = 10, 248.11 ± 79.13 s)]. And, the retention latency of the MPTP + vehicle-acute group was comparable with that of the MPTP + propofol-acute group (*p* = 0.894). Tukey’s MCT analysis indicated that both rats in the MPTP + vehicle-chronic group (*n* = 10) and MPTP + propofol-chronic group (*n* = 10) had obvious amnesia, with retention latencies of 147.84 ± 115.16 s and 48.88 ± 23.42 s, respectively [*p* = 0.028, *p* < 0.001 vs. rats in the sham + vehicle-chronic group (*n* = 10, 249.30 ± 82.45 s)]. Moreover, rats in the MPTP + propofol-chronic group showed more serious memory impairment than rats in the MPTP + vehicle-chronic group (*p* = 0.033).

### Neither acute nor chronic treatment with propofol can significantly change MPTP-induced decrease in TH immunoreactivity in the striatum

**Figure 4:**
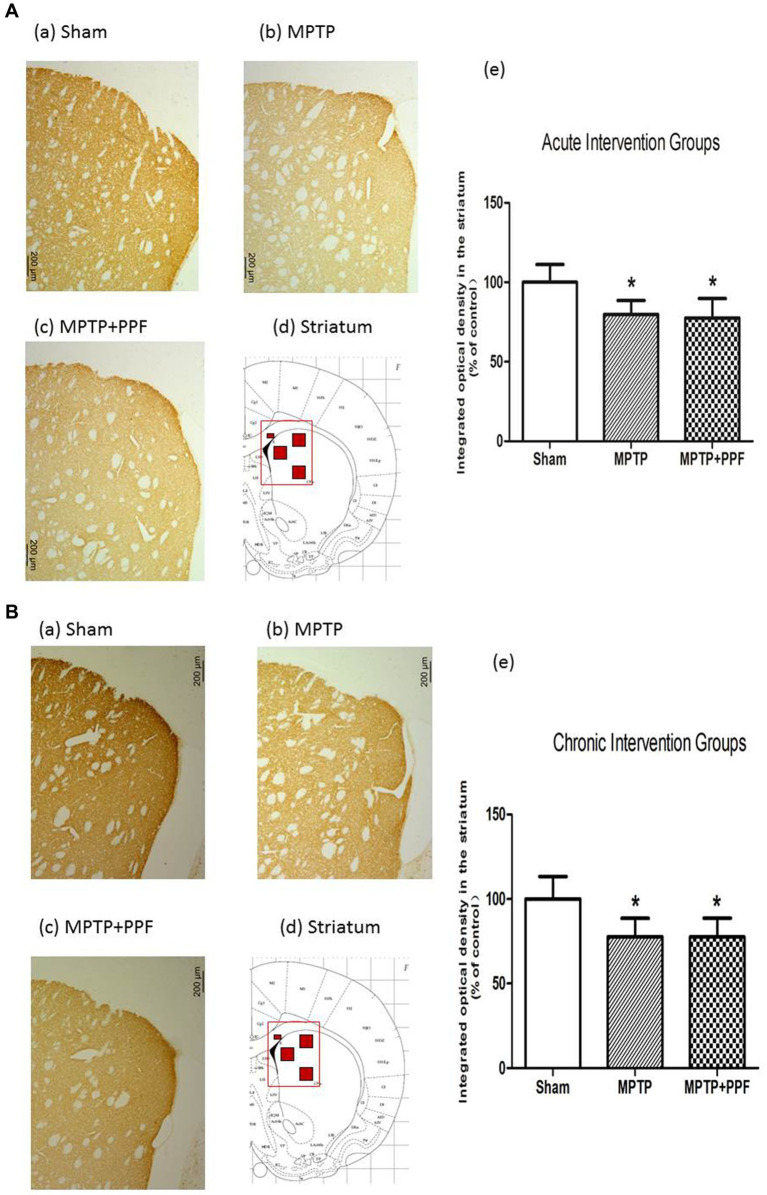
Tyrosine hydroxylase (TH)-positive neurons in the striatum of the acute **(A)** and chronic **(B)** intervention groups. **(a–c)** TH immunoreactivity in representative coronal sections. Magnification, 50×; bar, 200 μm. The rectangle in **d** indicates the area shown in **a–c**, and the small red squares inside the rectangle indicate the area used for measuring the optical density. **(e)** Quantitative results. Rats-treated with MTPT showed a significantly lower background-corrected TH immunoreactivity optical density in the striatum. The MPTP-induced decreases in TH immunoreactivity in the striatum were not changed by either acute or chronic propofol treatment ^*^p < 0.05 compared with the control (sham) group, *n* = 5 in each group.

Representative photomicrographs of immunostained brain sections are shown in Figure 4. TH immunoreactivity was observed in DAergic processes in the striatum. ANOVA showed that rats in the MPTP + vehicle group exhibited a lower background-corrected TH immunoreactivity optical density in the striatum (*p* < 0.05) compared to the sham + vehicle group. The MPTP-induced decreases in TH immunoreactivity in the striatum were not significantly changed by either acute or chronic propofol treatment (*p* > 0.05).

### Chronic treatment with propofol increases the imbalance of NR2A/NR2B ratio in the hippocampus in the MPTP-induced PD rat model

**Figure 5:**
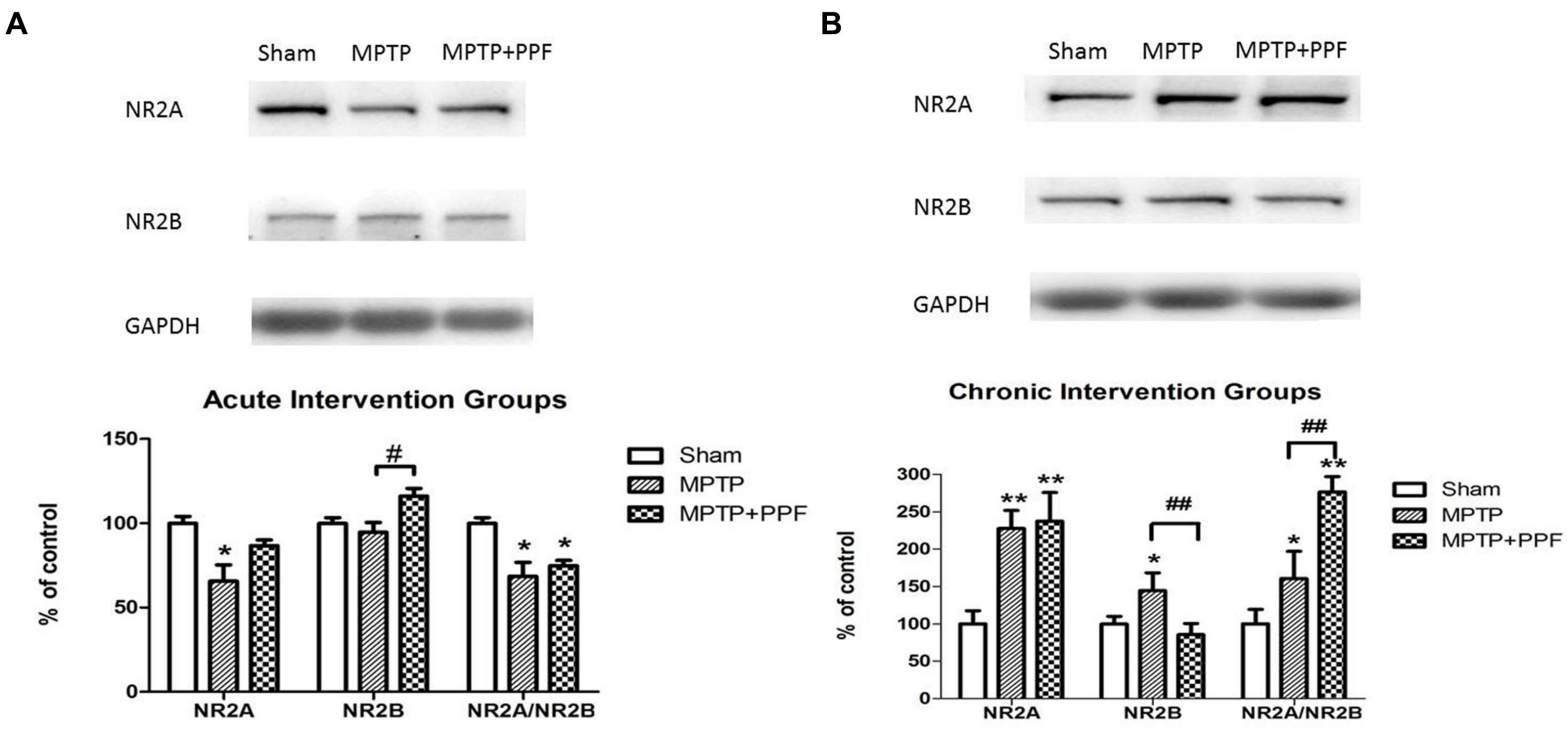
Western blot for detecting the distribution of hippocampal NMDA receptor NR2A and NR2B subunits in the acute **(A)** and chronic **(B)** intervention groups. Statistical analysis revealed that MPTP-lesioning caused an imbalance of NR2A/NR2B ratio when compared with the sham groups. And, chronic treatment with propofol increases the imbalance of NR2A/NR2B ratio in the hippocampus in the MPTP-induced PD rat model. ^*^p < 0.05 and ^**^p < 0.001 compared with the control (sham) group. ^#^*p* < 0.05 and ^##^*p* < 0.001 compared with the MPTP group, *n* = 5 in each group.

**Figure 6:**
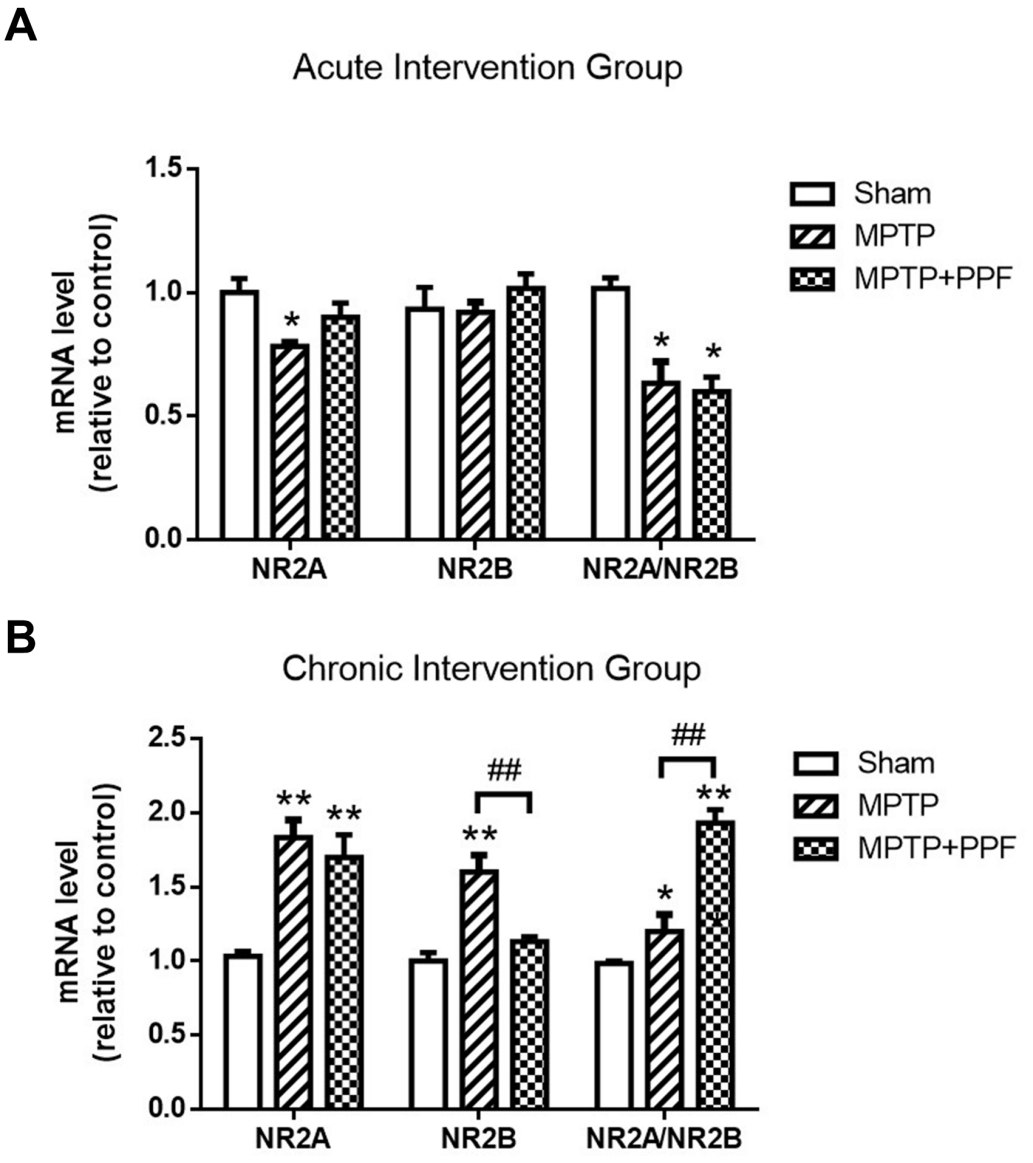
The expression levels of hippocampal NMDA receptor NR2A and NR2B mRNA in the acute **(A)** and chronic **(B)** intervention groups were quantified by RT-qPCR. In the chronic intervention groups, propofol increases the NR2A/NR2B mRNA ratio in the hippocampus in the MPTP-induced PD rat model. ^*^p < 0.05 and ^**^p < 0.001 compared with the control (sham) group. ^#^*p* < 0.05 and ^##^*p* < 0.001 compared with the MPTP group, *n* = 5 in each group.

The results were shown in Figures 5, 6. For the chronic intervention groups, it was observed that when compared with the sham group, the protein and mRNA levels of NR2A were significantly increased in both the MPTP + vehicle group and the MPTP + propofol group. The protein and mRNA levels of NR2B were elevated only in the MPTP + vehicle group, whereas no alteration observed in MPTP + propofol group.

Both the acute and chronic treatment experiments showed that MPTP-lesioning caused an imbalance of NR2A/NR2B ratio when compared with the sham groups. Furthermore, the chronic intervention groups showed that the MPTP + propofol group aggravated the increases of NR2A/NR2B ratio when compared with the MPTP + vehicle group.

## Discussion

There are three key findings of the present study. First, MPTP lesioning caused IA memory impairment, which was aggravated by chronic, but not acute, treatment with propofol at a dosage of 80 mg/kg/day. To our knowledge, this is the first evidence that propofol can worsen cognitive function in a PD rat model. Second, chronic treatment with propofol aggravated the imbalance of hippocampal NR2A/NR2B ratio in MPTP-lesioned rats. Third, neither acute nor chronic treatment with propofol altered the decreases of locomotor activity and DAergic denervation of the striatum in MPTP-lesioned rats.

Some studies suggest that propofol may have both pro-inflammatory and anti-inflammatory effects, depending on factors such as dosage, exposure time, animal disease mod and other variables. It has been proved that chronic treatment with propofol might improve cognitive function via attenuating the Aβ-induced mitochondria dysfunction and caspase activation in both aged and Alzheimer’s disease transgenic mice (Shao et al., 2014). However, increasing evidence suggests that propofol may possess neurotoxic effects. Yang et al. (2021) reported that propofol enhances the activation of astrocytes and increases levels of neuronal nitric oxide synthase, pro-inflammatory cytokines such as IL-6, and TNF-α, leading to inflammation in the hippocampus. In this study, we investigated the effects of propofol on cognitive function in a PD rat model. Although propofol is suggested to depress glutamatergic synaptic transmission and reduce potential excitotoxicity, the current results showed that chronic treatment with propofol did not alleviate, but aggravated the cognitive dysfunction in an MPTP-induced PD rat model. This could be due to differences in disease pathology or mechanisms. PD-related glutamate excitotoxicity might render NMDA receptors more vulnerable to propofol’s modulation, whereas propofol’s antioxidant properties could dominate in non-PD contexts.

NMDA receptor-mediated neurotransmission in the hippocampus is implicated in cognitive deficits in PD. Both the NR2A and NR2B subunits of NMDA receptor have been reported to play crucial roles in hippocampal LTP and memory process (Yokota et al., 2015; Belloso-Iguerategui et al., 2023). Several animal studies have observed that cognitive dysfunction is associated with reduced hippocampal NR2A and NR2B expression (Yokota et al., 2015; Zhang et al., 2015). However, other experiments indicated that the excessive up-regulation of NR2A and NR2B expression might contribute to cognitive dysfunction (Costa-Nunes et al., 2014; Dong et al., 2010). Therefore, the potential effects of the NR2A and NR2B levels on cognitive function are still controversial. It has been proposed that a correct balance between NR2A and NR2B at hippocampal synapses is critically important for LTP induction (Costa et al., 2012). Therefore, in the present study, we focused on the effects of propofol on the hippocampal NR2A, NR2B expression and NR2A/NR2B ratio in the MPTP-induced PD rats.

Many conflicting observations regarding the effects of propofol on NR2B expression and cognitive function have been reported previously. For instance, chronic treatment of propofol inhibited the excessive up-regulation of hippocampal NR2B expression caused by electroconvulsive therapy of depressed rats and improved spatial memory impairment (Dong et al., 2010). In the present experiment, the modulation effect of propofol on NR2B was associated with a more serious IA memory impairment in MPTP-lesioned rats. Propofol downregulated NR2B in the PFC of the developing rat brain and caused long-term cognitive dysfunction (Chen et al., 2019). The possible reasons for this discrepancy might be different ages, species of rats, and strategies for drug treatment.

It should be noted that the acute and chronic vehicle treated, MPTP lesioned rats showed distinct changes of NR2A and NR2B expression when compared, respectively, with the acute and chronic vehicle treated, sham lesioned rats. However, the imbalances of NR2A/NR2B ratio were observed in all MPTP lesioned groups. The current results support the hypothesis that a correct balance of NR2A/NR2B ratio is crucial for normal cognitive function. Moreover, from the chronic intervention groups, it was observed that the hippocampal NR2A/NR2B ratio was significantly increased in the MPTP-lesioned rats and further increased by chronic propofol treatment, when compared with the NR2A/NR2B ratio of the sham-lesioned group. The chronic treatment of propofol aggravated the imbalance of NR2A/NR2B ratio, which might contribute to the aggravation of cognitive impairment in MPTP-induced PD rats. Our current data demonstrate a correlation, not direct causation, between NR2A/NR2B dysregulation and cognitive impairment. Future studies should probe whether this imbalance is a driver or consequence of synaptic dysfunction.

Propofol exerts complex and region-specific effects on the dopaminergic circuitry across various brain areas. Chronic propofol exposure led to a significant reduction in dopaminergic neurons in the striatum, along with decreased locomotor activity (reduced travel distance and mobile time) compared to control mice (Chen et al., 2020). Our study observed similar findings. Both acute and chronic treatment with propofol did not significantly change the DAergic denervation of the striatum or influence the locomotor activity in the MPTP-induced PD rat model. Although propofol has been demonstrated to elevate dopamine concentrations in the nucleus accumbens (NAc) of normal mice (Zhu et al., 2023), our findings suggest this effect may not extend to the striatum in MPTP-lesioned rats. This regional specificity could explain the lack of motor improvement, as striatal (rather than accumbens) dopamine depletion is primarily responsible for PD motor symptoms. Future research should focus on elucidating propofol’s differential effects on dopaminergic systems across distinct brain regions.

### Limitations and future perspectives

There are several limitations of the current study. First, only IA test was detected in the current study, it is still not clear whether this phenomenon is task-specific. Future studies with additional behavioral tests (e.g., Morris water maze for spatial memory, novel object recognition for non-aversive memory) are needed to validate and extend our findings. Second, since the lack of a group in which the propofol alone was chronically administered, it is not clear whether there is any interaction between propofol and MTPT. In future studies, it is important to establish a propofol-only group. Third, in clinic, propofol is widely used for the maintenance of general anesthesia. To better evaluate the effect of propofol on cognitive function of surgical patients with PD, further study is needed to detect the effect of continuous intravenous infusion of propofol on MPTP-induced PD rats. Last, in the present study, total hippocampal NR2A and NR2B subunit levels were detected. It might be more convincing to detect the NR2A and NR2B subunit levels at hippocampal synapses.

## Conclusion

The current findings indicate that propofol administration (acute or chronic) did not significantly alter locomotor deficits or striatal dopaminergic denervation in MPTP-lesioned rats. However, chronic propofol treatment exacerbated MPTP-induced inhibitory avoidance (IA) memory impairment and aggravated the imbalance of hippocampal NR2A/NR2B ratio in MPTP-lesioned rats.

## Data Availability

The raw data supporting the conclusions of this article will be made available by the authors, without undue reservation.
